# Dequalinium blocks macrophage-induced metastasis following local radiation

**DOI:** 10.18632/oncotarget.4826

**Published:** 2015-08-06

**Authors:** Michael Timaner, Rotem Bril, Orit Kaidar-Person, Chen Rachman-Tzemah, Dror Alishekevitz, Ruslana Kotsofruk, Valeria Miller, Alexander Nevelsky, Shahar Daniel, Ziv Raviv, Susan A. Rotenberg, Yuval Shaked

**Affiliations:** ^1^ Department of Cell biology and cancer science, Rappaport Faculty of Medicine, Technion, Haifa, Israel; ^2^ Department of Radio-Oncology, Rambam Health Care Campus, Haifa, Israel; ^3^ Department of Chemistry and Biochemistry, Queens College of the City University of New York, Flushing, NY, USA

**Keywords:** radiotherapy, macrophages, colon cancer, tumor microenvironment

## Abstract

A major therapeutic obstacle in clinical oncology is intrinsic or acquired resistance to therapy, leading to subsequent relapse. We have previously shown that systemic administration of different cytotoxic drugs can induce a host response that contributes to tumor angiogenesis, regrowth and metastasis. Here we characterize the host response to a single dose of local radiation, and its contribution to tumor progression and metastasis. We show that plasma from locally irradiated mice increases the migratory and invasive properties of colon carcinoma cells. Furthermore, locally irradiated mice intravenously injected with CT26 colon carcinoma cells succumb to pulmonary metastasis earlier than their respective controls. Consequently, orthotopically implanted SW480 human colon carcinoma cells in mice that underwent radiation, exhibited increased metastasis to the lungs and liver compared to their control tumors. The irradiated tumors exhibited an increase in the colonization of macrophages compared to their respective controls; and macrophage depletion in irradiated tumor-bearing mice reduces the number of metastatic lesions. Finally, the anti-tumor agent, dequalinium-14, in addition to its anti-tumor effect, reduces macrophage motility, inhibits macrophage infiltration of irradiated tumors and reduces the extent of metastasis in locally irradiated mice. Overall, this study demonstrates the adverse effects of local radiation on the host that result in macrophage-induced metastasis.

## INTRODUCTION

Ionizing radiation is a major treatment strategy for a variety of cancers, such as brain, rectal, cervix and breast cancers among others. Radiation directly affects tumor cells by inducing extensive DNA damage and subsequently cell death [1]. However, DNA and tissue/cell repair can take place, leading to tumor recovery and relapse especially in the irradiated field [2–4]. Radiation-induced damage in normal surrounding tissue promotes an inflammatory response and angiogenesis as part of the healing process [5]. Recent studies by Brown and colleagues demonstrated that recurrence following radiotherapy of brain tumors is associated with the recruitment of bone marrow derived cells (BMDCs) to the irradiated tumor site, and that such cells contribute to angiogenesis and tumor resistance [6, 7]. They showed that HIF-1 induction in the irradiated tumors promotes BMDC influx, and that therapeutic blockade of BMDC trafficking prevents or delays tumor recurrence [6].

The contribution of BMDCs to tumor re-growth has been mostly studied following systemic administration of cytotoxic drugs. We and others recently demonstrated that the recruitment of BMDCs, in particular endothelial progenitor cells (EPCs), to tumors following therapy enhances angiogenesis leading to tumor regrowth [8, 9]. Myeloid cells such as Tie-2 expressing monocytes have also been shown to support tumor angiogenesis and re-growth following therapy with vascular disrupting agents [10]. Importantly, the mobilization and homing of BMDCs following such therapies are mostly related to the responses of the host to therapy since these effects were also observed in non-tumor bearing mice treated with the cytotoxic agent [11].

The invasion and colonization of BMDCs at the tumor site following therapy can also affect metastasis. Previously, we demonstrated that MMP9 secreted by BMDCs in response to chemotherapy promotes metastasis. The selective lack of MMP9 expression in BMDCs resulted in extended survival of chemotherapy-treated mice bearing pulmonary metastases [12]. In addition, Daenen et al. showed that pre-treatment with chemotherapy prior to tumor inoculation significantly enhances lung metastasis in mice [13]. Thus, mobilized BMDCs may play a major role in the metastatic process, especially following chemotherapy [14]. In the context of radiation, the secretion of MMP9 by BMDCs leads to an induction in angiogenesis and tumor growth [15]. In addition, it has been demonstrated that in relapsing tumors from radiotherapy, the tumor microenvironment triggers the secretion of hypoxic pro-metastatic-related factors which can support tumor aggressiveness and metastasis [16, 17]. However, the host effects of the normal tissue generated in response to radiation, their impact on tumor cells, and their metastatic properties have not been fully explored in this context.

Radiotherapy induces tumor-promoting changes within the tumor microenvironment directly affecting both tumor and host cells, leading to aggressive tumor characteristics [18–20]. Nguyen et al. demonstrated that gene expression within tumors of whole body irradiated mice favors an aggressive phenotype compared to tumors of non-irradiated mice [20]. These results highlight the role of host cells exposed to radiation and their effect on tumor fate [21]. In this study we investigated whether local radiotherapy induces a pro-metastatic host response, in a similar manner to systemic chemotherapy. We found that the exposure of ‘naïve’ mice to local radiation enhances the metastatic phenotype of tumors, an effect partially mediated by macrophages which home to radiotherapy-treated tumors. We found that the blockade of macrophage recruitment to the irradiated tumor site by dequalinium-14 (DECA), a potent anti-tumor agent [22, 23], inhibits radiotherapy-induced metastasis. Our results therefore suggest that blocking host effects induced by radiation may improve therapy outcome and inhibit metastatic potential.

## RESULTS

### Host response to local radiation promotes systemic, but not local angiogenesis

**Figure 1:**
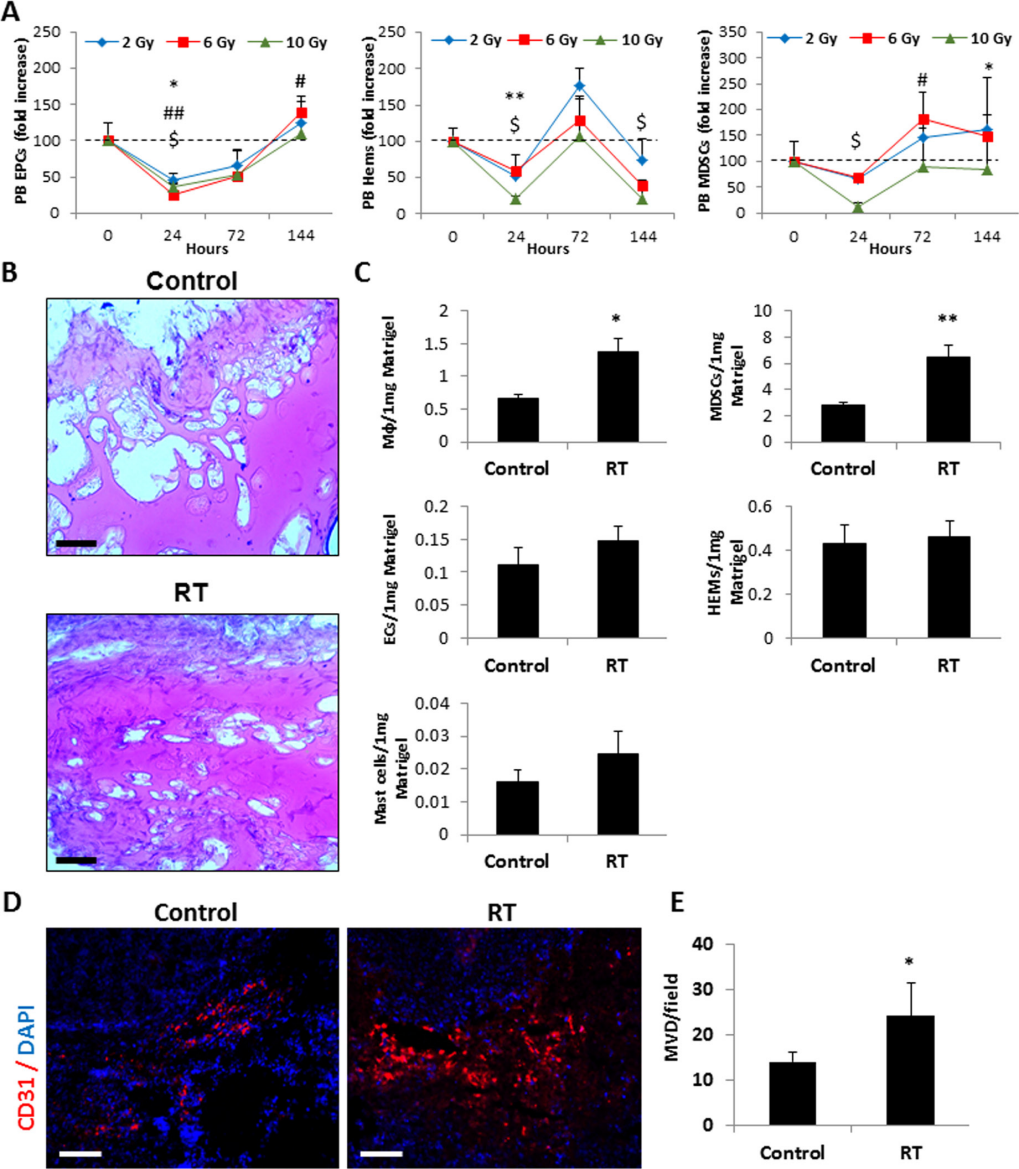
Host response to single dose local radiation promotes BMDC mobilization and colonization in Matrigel plugs **A.** Eight-to-ten week old non-tumor bearing BALB/c mice were exposed to a single dose of local radiation (RT) (2 Gy, 6 Gy and 10 Gy) in the abdominal cavity. Blood was drawn from the retro-orbital sinus at baseline, 24, 72 and 144 hours after radiation. The levels of viable circulating endothelial progenitor cells (EPCs), hemangiocytes (Hems) and myeloid derived suppressor cells (MDSCs) in peripheral blood (PB) were evaluated as described in Materials and Methods (*n* = 4–5 mice/group). Statistical significance (*p* < 0.05) between baseline and 2 Gy (*), 6 Gy (#) and 10 Gy ($) local radiation is shown. **B-C.** Matrigel plugs containing 10% plasma collected from untreated control (Control) or locally irradiated mice (24 hours following 2Gy radiation) (RT) were implanted into the flanks of 8–10-week-old BALB/c mice. After 10 days, the plugs were removed and processed for histology and flow cytometry. Matrigel cryosections were stained with H&E (B). Matrigel plugs were prepared as single cell suspensions and the extracted cells were immunostained for various BMDC populations including macrophages (Mφ), MDSCs, endothelial cells (ECs), hemangiocytes (HEMs), and Mast cells. Results are presented as the number of cells per 1 mg Matrigel (C). Scale bars = 100 μm. **D-E.** Eight-to-ten week old SCID mice were orthotopically implanted with SW480 tumors and either left untreated (control) or exposed to 2Gy RT. After 72 hours, the tumors were harvested, sectioned and immunostained for endothelial cells (CD31, red). Nuclei were stained with DAPI (blue) (D). Scale bars = 200 μm. The number of microvessel structures per field was counted (*n* > 10 fields/group) (E). *0.05 > *p* > 0.01; **0.01 > *p* > 0.001; ***p < 0.001.

Previous studies have reported that tumors exposed to local radiation exhibit an increased infiltration of CD11b-positive myelomonocytic cells at the irradiated tumor site, promoting systemic angiogenesis [6, 7]. We asked whether the nature of such results can be achieved when evaluating solely the host effects in response to radiation by using non-tumor bearing mice, as previously shown for chemotherapy [12]. To do so, we examined the mobilization of different types of angiogenesis-promoting BMDCs to peripheral blood in naïve mice that were exposed to escalating dosages of radiation. Figure 1A shows the dynamic changes in the levels of viable circulating EPCs (CEPs), hemangiocytes and myeloid-derived suppressor cells (MDSCs) in peripheral blood of mice exposed to 2 Gy, 6 Gy and 10 Gy radiation. All cell types decreased significantly within the first 24 hours. After 72 hours, the levels of hemangiocytes and MDSCs increased in the peripheral blood of mice exposed to 2 Gy, 6 Gy, but not 10 Gy radiation, compared to non-irradiated mice. Based on these results, we chose to focus on the host response effects to local radiation using the clinically relevant fraction daily dose of 2 Gy [1], which does not result in a significant cell suppression found in the higher radiation doses.

To further evaluate the host response to radiation, Matrigel plugs containing plasma obtained from mice exposed to 2 Gy radiation for 24 hours were injected into the flanks of naïve BALB/c mice. Ten days later, the plugs were removed and further analyzed. Histological analysis revealed that plugs containing plasma from irradiated mice exhibited a significant increase in the colonization of host cells compared to plugs containing plasma from control mice (Figure 1B). Specifically, the numbers of MDSCs and macrophages colonizing the Matrigel plugs were significantly higher whereas all other BMDCs tested, including hemangiocytes and endothelial cells did not significantly change between the two groups (Figure 1C).

To determine whether the host response to local radiation promotes local angiogenesis independent of radiation-induced tumor hypoxia as previously reported [24], we employed endothelial cell migration, microvessel sprouting from aortic rings, and HUVEC tube formation assays, comparing the effect of plasma from irradiated and control mice. No significant differences in endothelial cell activity were detected (Supplemental Figure 1A-1C). However, a significant increase in tumor angiogenesis was observed in SW480 tumors three days after they were exposed to a single dose of 2 Gy radiation (Figure 1D–1E). Taken together, these results suggest that the host response to 2 Gy radiation affects the mobilization of specific BMDCs to peripheral blood which may contribute to tumor angiogenesis, an effect which is unlikely related to local sprouting angiogenesis.

### Plasma from locally irradiated mice induces migration and invasion of tumor cells

**Figure 2:**
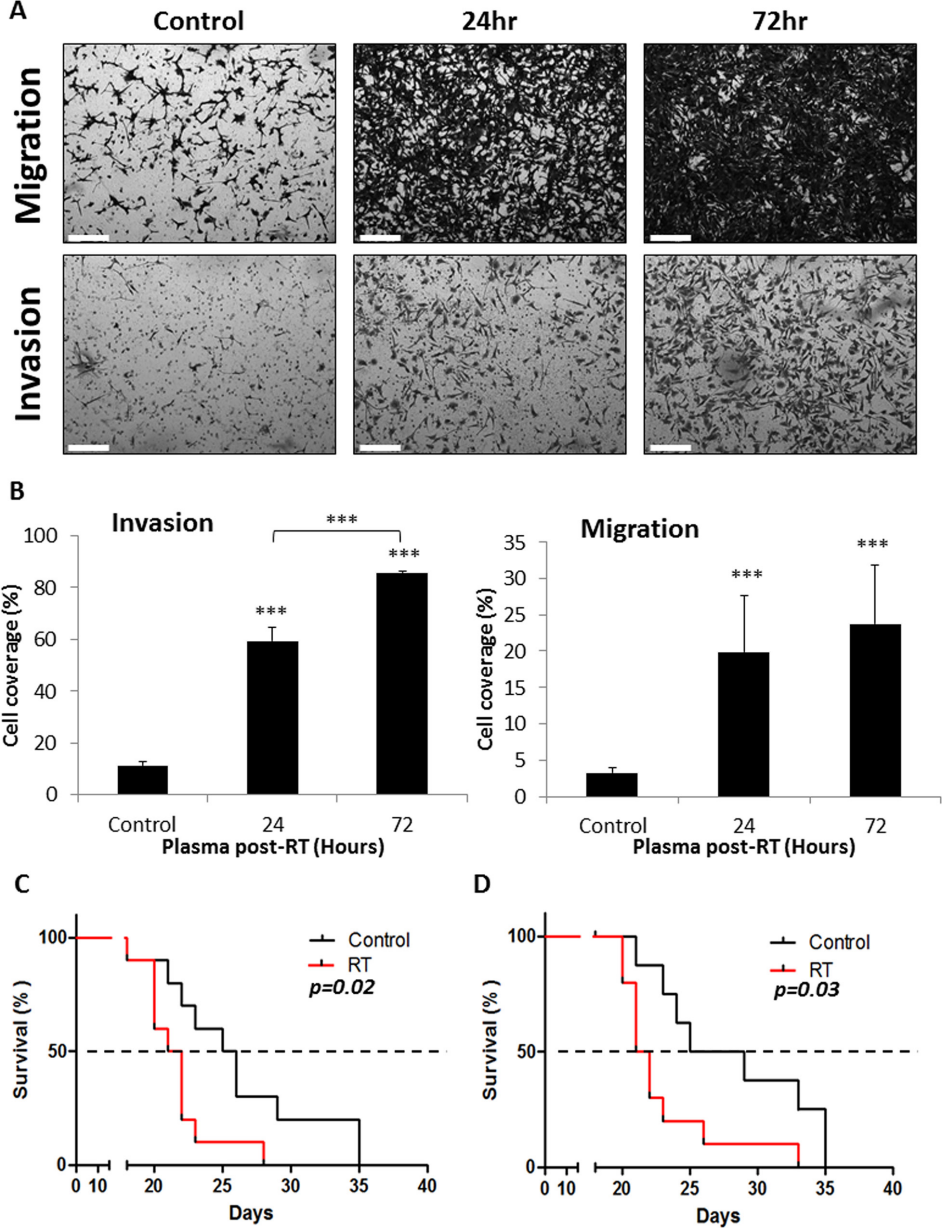
Host response to local radiation promotes tumor cell metastatic properties **A-B.** Eight-to-ten week old non-tumor bearing BALB/c mice were exposed to a single dose of 2 Gy radiation in the abdominal cavity. Plasma was collected at baseline (control), 24, and 72 hours after RT and used in the modified Boyden chamber assay to assess migration and invasion properties of murine colorectal carcinoma cells, CT26. Images were captured using the Leica CTR 6000 microscope system (Leica Microsystems) (A), and the number of CT26 cells invading or migrating through the membrane were quantified (B) (Scale bars = 200 μm). **C-D.** Mice were either left untreated (control) or pre-exposed to 2 Gy RT in the abdominal cavity (C) or intraperitoneally injected with 100 μl of plasma from untreated or 2 Gy irradiated mice (D) Twenty four hours later, mice were injected with 0.5 × 10^6^ CT26 cells and survival rates were assessed over time (*n* = 9–10 mice/group).***p < 0.001.

We have recently shown that the host response to different chemotherapy drugs induces pro-tumorigenic and pro-metastatic effects, by means of promoting tumor cell invasion and migration [12]. We therefore sought to determine whether radiotherapy promotes comparable effects to those reported for chemotherapy. To test this, 8–10 week old BALB/c mice were exposed to a single dose of 2 Gy radiation to the abdominal cavity. Plasma was extracted at different time points (24–144 hours), and the samples were applied to a Boyden chamber assay to examine the migration and invasion properties of murine CT26 and human HCT116 colon carcinoma cells. The results in Figure 2A–2B and Supplemental Figure 2A-2B show that migration and invasion of the cancer cells were significantly increased in the presence of plasma from mice exposed to radiotherapy when compared to plasma from control mice. Of note, no change in tumor cell migration and invasion was observed when the plasma was placed directly on the tumor cells (Supplemental Figure 2C), demonstrating that these effects were due to chemo-attraction mechanisms and not only direct activation of cancer cells.

To further evaluate the metastatic properties of tumor cells induced by the host response to radiation, CT26 cells were intravenously injected into the tail vein of mice previously exposed to radiation in the abdominal cavity, and survival was monitored over time. As seen in Figure 2C, mice pre-exposed to radiation succumbed to pulmonary metastases earlier than control mice (*p* = 0.02). To rule out the possibility that radiation promotes metastasis by directly affecting the host tissue, we injected naïve mice with plasma obtained from irradiated and control mice. Twenty four hours later, CT26 cells were injected into the tail vein, and survival was monitored over time. The results in Figure 2D demonstrate that the mortality rate of mice injected with plasma obtained from irradiated mice was higher than that of mice injected with control plasma (*p* = 0.03). Taken together, our results indicate that local radiation induces a host response that in turn enhances migration and invasion of tumor cells, promoting their seeding at metastatic sites, and increasing mortality rate in mice bearing experimental lung metastases.

### Macrophages from locally irradiated mice contribute to the metastatic properties of tumor cells

**Figure 3:**
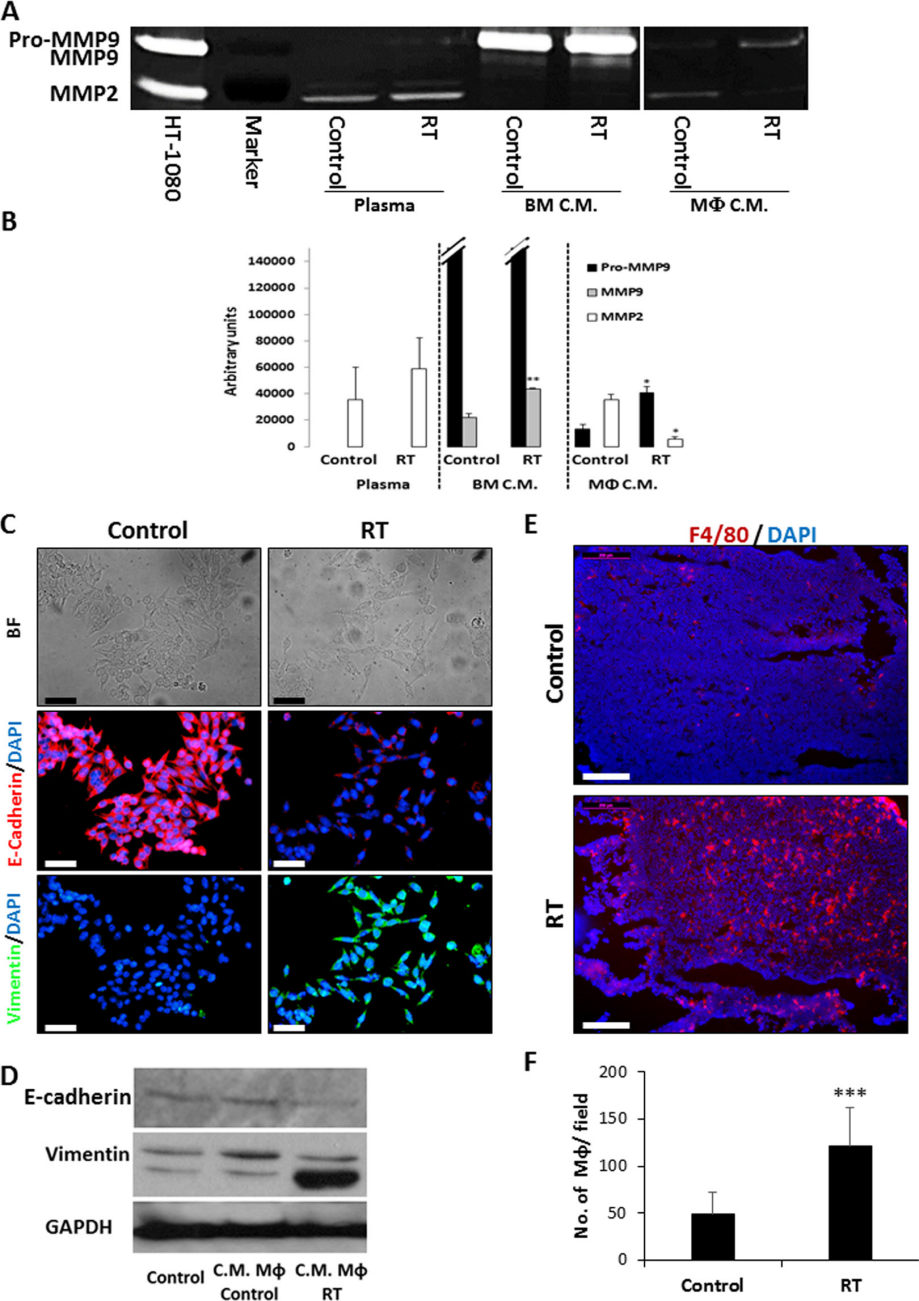
Conditioned medium of macrophages collected from locally irradiated mice promotes metastasis **A.** The activities of MMP2 and MMP9 in plasma extracted from control and 2 Gy locally irradiated (RT) mice as well as in conditioned medium (C.M.) of BMDCs and macrophages derived from these mice were assessed by zymography. Conditioned medium of HT1080 cells was used as a positive control. Experiments were performed in triplicate. **B.** The expression levels of MMP2, pro-MMP9, and active MMP9 were determined by densitometry analysis of the zymography gels. **C-D.** SW480 cells were cultured for 24 hours in conditioned medium of macrophages obtained from control or locally-irradiated mice (RT). To determine the extent of epithelial-to-mesenchymal transition (EMT), cells were stained with anti-E-cadherin (red), anti-vimentin (green) antibodies, and counterstained with DAPI (C), or cell lysates were analyzed by Western Blot (D). Images were captured using the Leica CTR 6000 microscope system. Representative bright field (BF) and immunofluorescent images are shown. Scale bars = 50 μm. **E.** Eight-to-ten week old SCID mice were orthotopically implanted with SW480 tumors and either left untreated (control) or exposed to 2Gy RT. After 72 hours, the tumors were harvested, sectioned and immunostained for macrophages (F4/80, red). Nuclei were stained with DAPI (blue). GPDH served as a loading control. Scale bars = 200 μm. **F.** The number of macrophages per field were counted (*n* > 10 fields/group). *p < 0.05; **p < 0.01; ***p < 0.001.

Previous studies have shown that BMDCs colonizing chemotherapy-treated tumors highly express MMP9. This may provide an explanation for tumor cell dissemination from the primary tumor due to epithelial to mesenchymal transition (EMT) observed following chemotherapy [12, 15]. To determine whether radiation affects MMPs expression, we assessed the levels of MMP2 and MMP9 in the plasma of mice exposed to 2 Gy radiation as well as in conditioned medium (CM) of BMDCs and peritoneal macrophages collected from these mice. The levels of MMP2 and MMP9 were similar in the plasma of irradiated and control mice. However, the level of MMP9 was increased by 2-fold in the CM of bone marrow cells from irradiated mice (*p* < 0.01). In addition, pro-MMP9 was highly elevated and MMP2 was reduced in the CM of macrophages from irradiated mice (*p* < 0.05, Figure 3A–3B).

Next, we tested the ability of the CM of macrophages obtained from irradiated mice to induce EMT. To this end, SW480 cells, which are usually used for EMT assay [25], were cultured with CM of peritoneal macrophages obtained from control and irradiated mice. After 24 hours of incubation, cells were fixed and immunostained for epithelial (E-cadherin) and mesenchymal (vimentin) specific markers. The results in Figure 3C–3D and Supplemental Figure 3 show that CM of macrophages obtained from irradiated mice promoted EMT of SW480 cells based on their spindle-like morphology, loss of E-cadherin expression and gain of vimentin expression. Importantly, macrophages infiltrated orthotopically implanted SW480 tumors of mice exposed to local radiation to a greater extent than tumors of control mice (Figure 3E–3F). Collectively, these results provide additional support to the notion that the host response to a single dose of radiation promotes metastatic properties of tumor cells, and that such effects might be linked to macrophages.

### Local radiation increases metastasis of colon carcinoma tumors

**Figure 4:**
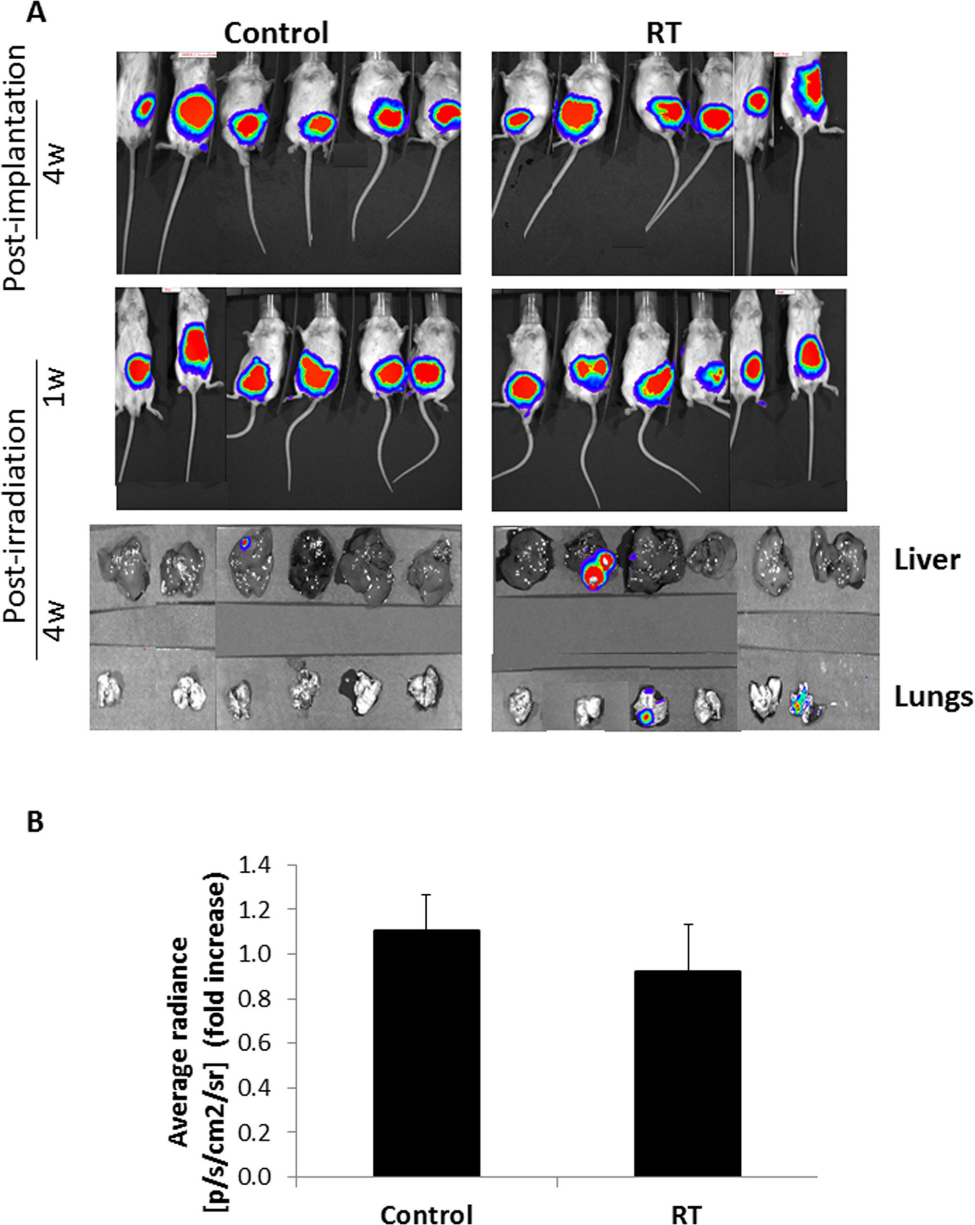
Local radiation at a single 2 Gy dose induces metastasis of orthotopic colon carcinoma tumors **A.** Eight-to-ten week old SCID mice were orthotopically implanted with SW480 tumors expressing luciferase. After 4 weeks, the mice were either irradiated in the abdominal cavity (RT) at a dose of 2 Gy or left untreated (Control). Tumor growth was monitored using IVIS *in vivo* imaging system. At end point (4 weeks later), liver and lungs were removed and examined for metastases. Shown are IVIS images of the mice at 4 weeks post-implantation, 1 week post radiation, and liver and lungs at end point. **B.** Tumor growth was quantified by calculating the change in average radiance of tumors from irradiated mice when compared to tumors from control mice, 1 week following radiation (*n* = 6 mice/group).

To further assess the pro-metastatic effects of radiation, we evaluated metastasis of SW480 tumors expressing luciferase orthotopically implanted into the cecum of SCID mice. SW480 tumor cells, in this experimental design, rarely metastasize to other organs as opposed to other colon carcinoma cells tested (see Supplemental Table 1). To this end, 1–3 mm^3^ fragments of SW480 subcutaneous tumor tissues were sutured to the cecum of SCID mice, and 4 weeks later, when detectable tumors were observed by *in vivo* imaging system (IVIS), mice were exposed to 2 Gy radiation in the tumor area. Four weeks later, lungs and livers were examined for the presence of visible metastases. Figure 4 demonstrates that while a single dose of 2 Gy radiation resulted in a non-significant reduction in tumor growth one week after radiotherapy, the irradiated mice exhibited an increased number of macro-metastatic lesions in the lungs and liver when compared to control mice (3/6 and 1/6, respectively). This experiment was repeated a number of times in the same manner, and comparable results were obtained (see Supplemental Table 2). The accumulative results from all experiments performed in Supplemental Table 2 reached statistical significance (*p* < 0.001) using the Fisher Exact Test, when comparing overall incidence of liver and lung metastases between control and irradiated mice. Furthermore, similar results were observed when CT26 metastatic cells were orthotopically implanted in 8–10 week old BALB/c mice. Namely, a substantial increase in the number of metastatic lesions in the liver and lungs were observed in mice that underwent radiotherapy when compared to control untreated mice (Supplemental Figure 4). Thus, these *in vivo* experiments indicate that a single dose of 2 Gy radiation increases the metastatic properties of tumor cells, similar to findings from the *in vitro* experiments.

### Macrophages mediate radiotherapy-induced metastasis in mice

**Figure 5:**
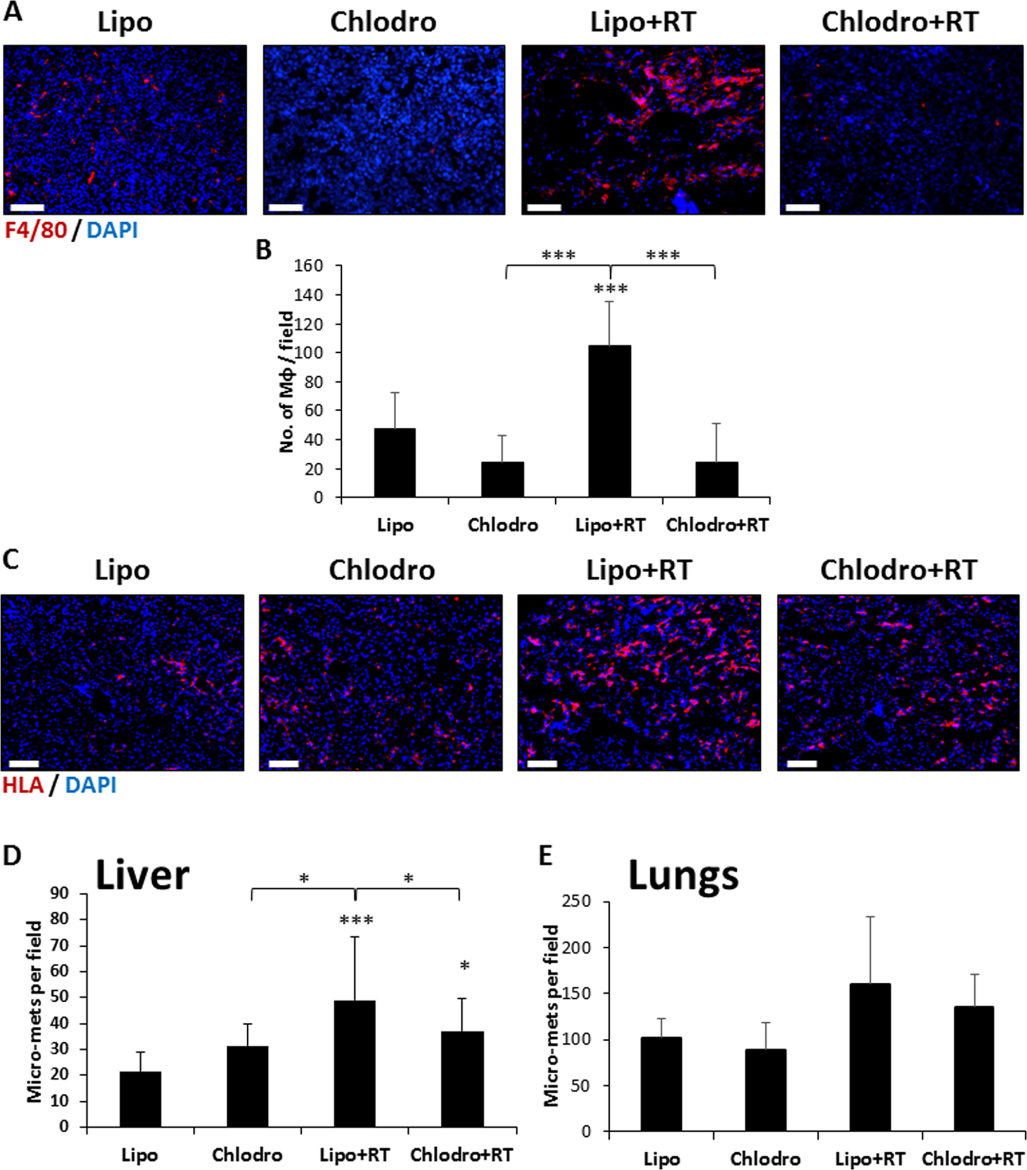
Macrophage-depleted irradiated mice exhibit reduced micrometastasis Eight-to-ten week old SCID mice were orthotopically implanted with SW480 tumors. After 4 weeks, the mice were either injected with empty liposomes (Lipo) or liposomes containing Clodronate to deplete macrophages (Clodro). After 24 hours, mice were either exposed to 2 Gy radiation (RT) in the tumor area or left untreated (Control) (*n* = 5 mice/group). After an additional 4 weeks the mice were sacrificed and tumors, lungs and liver were processed for histopathology analysis. **A.** Tumor sections were stained for macrophages (F4/80, red). Nuclei were counterstained with DAPI (blue). Scale bars = 200 μm. **B.** The number of macrophages per field were counted (*n* > 10 fields/group). **C-E.** Liver and lung sections were stained with anti-human HLA (red), and nuclei were counterstained with DAPI (blue) (C). The number of metastasis in the liver (D) and the lungs (E) were counted per field (*n* > 10 fields/group). Scale bars = 200 μm. *0.05 > *p* > 0.01; ***p < 0.001.

Our findings that local radiation enhances the recruitment of macrophages to the tumor as well as increases MMP9 expression in these cells suggest a role for macrophages in promoting metastasis following radiotherapy. To further investigate the role of macrophages in promoting metastasis *in vivo*, we orthotopically implanted SW480 tumor fragments to the cecum of SCID mice. After 4 weeks, mice were depleted of macrophages by injecting them with clodronate-liposomes and 24 hours later, the mice were exposed to a single dose of 2 Gy radiation. Tumors were allowed to grow for an additional 4 weeks, at which point they were removed, and lungs and liver were harvested and analyzed for metastasis. Immunohistochemistry analysis of the excised tumors demonstrated efficient macrophage depletion in clodronate-injected mice when compared to empty-liposomes injected mice (Figure 5A–5B). In addition, irradiated mice exhibited a significant increase in micro-metastatic lesions in the liver, compared to control untreated mice. Interestingly, this effect was significantly reduced in the irradiated mice that had been depleted of macrophages (Figure 5C–5D, and Supplemental Figure 5). The same pattern was observed when assessing lung metastases, however, the differences did not reach statistical significance (Figure 5E). Overall, our results further suggest that macrophages contribute to metastatic spread in mice exposed to single dose radiation.

### Dequalinium-14 reduces metastasis in irradiated mice by inhibiting the motility and colonization of macrophages in treated tumors

**Figure 6:**
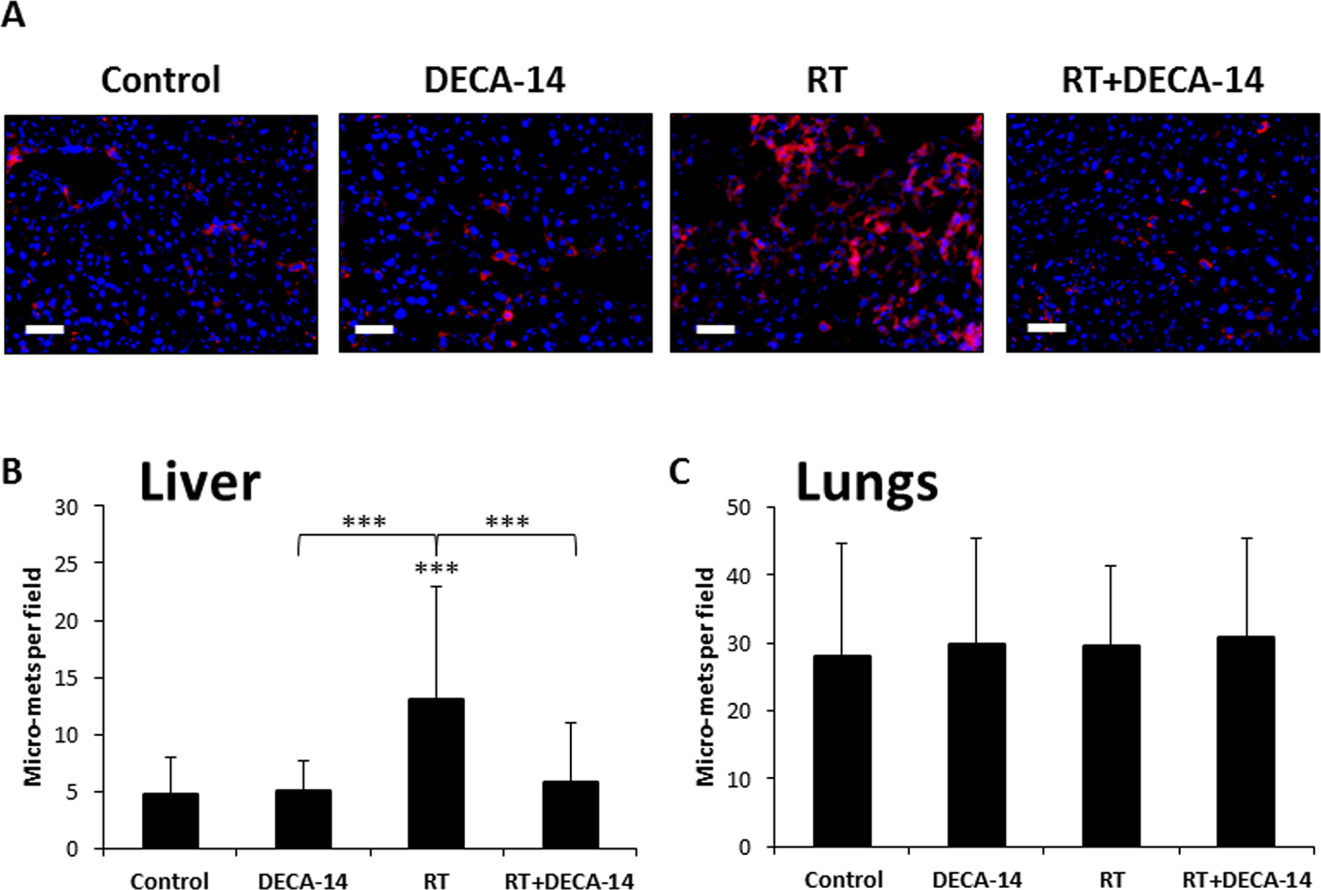
Dequalinium-14 (DECA-14) inhibits radiation-induced micrometastasis Eight-to-ten week old SCID mice were orthotopically implanted with SW480 tumors. After 4 weeks, the mice were peritoneally injected with 2.5mg/kg DECA-14, and after 24 hours, mice were either irradiated in the tumor area (RT) or left untreated (Control) (*n* = 5–6 mice/group). Four weeks later, the mice were sacrificed, and lungs and livers were removed and processed for histopathology analysis. **A.** Liver and lung sections were stained with anti-human HLA (red), and nuclei were counterstained with DAPI (blue). **B-C.** Micrometastasis counts per field in liver (B) and lungs (C) are presented. Scale bar = 100 μm. ***p < 0.001.

Dequalinium (DECA) is a potent anti-tumor agent, shown to selectively accumulate in cancer cells [22, 23]. It is a specific inhibitor of protein kinase C (PKC), a serine/threonine protein kinase which is essential for cell-cell adhesion, proliferation and metastasis [26, 27]. Earlier studies indicated that DECA-14 inhibited motility and invasiveness of human and murine melanoma cells [28, 29]. Based on its properties, we asked whether DECA-14 counteracts the pro-metastatic host response to radiation. To do so, mice were orthotopically implanted with SW480 colon tumors as above, and four weeks later they were administrated with 2.5 mg/kg DECA-14. Twenty four hours later the mice were locally irradiated and tumor growth was monitored over time using IVIS. This experiment revealed that DECA-14 inhibited primary tumor growth albeit not significantly, in the presence or absence of a single dose of radiation (Supplemental Figure 6). Histopathology analysis of the liver 4 weeks after mice were irradiated revealed that the significant increase in micro-metastasis in irradiated mice was dramatically reduced in irradiated mice administered with DECA-14. However, these effects were not observed in the lungs (Figure 6A–6C and Supplemental Figure 7).

**Figure 7:**
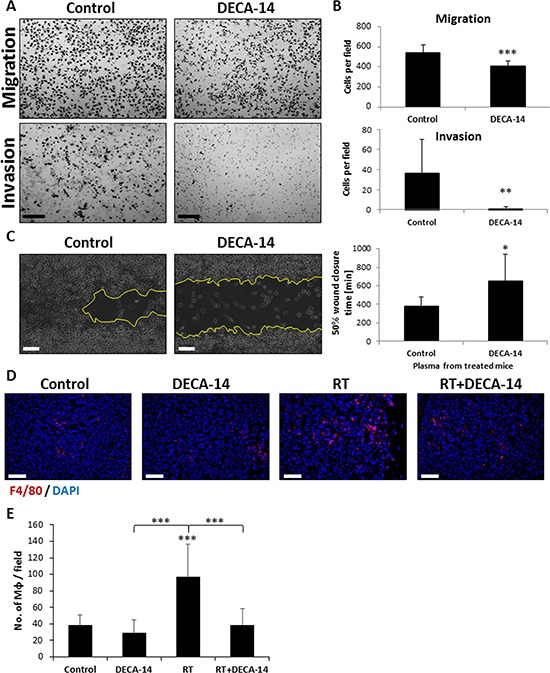
DECA-14 inhibits migratory properties of macrophages *in vitro* and *in vivo* **A-B.** The invasion and migration properties of J774 macrophages were assessed in the presence or absence of DECA-14 (100 nM) using the Boyden chamber assay. Images of the membranes were captured (A) and the number of cells per field was counted (B). Scale bars = 200 μm. **C.** A scratch wound assay was performed on J774 cells cultured in the presence of plasma obtained from mice 24 hours after they were treated with DECA-14, or from control mice. The percentage of wound closure time was evaluated and plotted using ImagePro Premier software. Representative images of 600 min time-point are presented. **D–E.** Eight-to-ten week old SCID mice were orthotopically implanted with SW480 tumors. After 4 weeks, the mice were intraperitoneally injected with 2.5 mg/kg DECA-14, and 24 hours later mice were either irradiated in the tumor area (RT) or left untreated (Control)(*n* = 5–6 mice/group). Three days later, tumors were harvested and (D) immunostained for macrophages (F4/80, red). Nuclei were stained with DAPI (blue). Scale bars = 100 μm. (E) The number of macrophages per field were counted (*n* > 10 fields/group). *0.05 > *p* > 0.01; **0.01 > *p* > 0.001; ***p < 0.001.

To investigate the mechanism by which DECA-14 reduces metastasis in irradiated mice, we assessed PKC activity in macrophages collected from irradiated mice. The results in Supplemental Figure 8A show that PKC activity was significantly reduced in macrophages from mice that had been treated with DECA-14 alone. However, in irradiated mice, no difference in PKC activity was observed in the presence or absence of DECA-14. We next determined the levels of MMP-9 and MMP-2 in conditioned medium of macrophages from the same groups as above. No substantial differences in the levels of MMP-9 and MMP-2 activities were observed in any of the groups tested as assessed by zymography (Supplemental Figure 8B). We therefore asked whether DECA-14 affects macrophage motility, as previously shown for other cells [28, 29]. To test this, J774 macrophages were seeded in a modified Boyden chamber and the effect of DECA-14 on their migration and invasion properties was evaluated and compared to the untreated control group. As shown in Figure 7A–7B, DECA-14 (100 nM) significantly inhibited migration and invasion of J774 macrophages. In addition, plasma from mice treated with DECA-14 suppressed motility of J774 macrophages as assessed by a scratch wound assay (Figure 7C). Moreover, a significant reduction in the number of macrophages colonizing primary orthotopic SW480 tumors of irradiated mice treated with DECA-14 was observed when compared to the number of macrophages in tumors from irradiated mice (Figure 7D–7E). Taken together, our results suggest that DECA-14 inhibits macrophage infiltration of irradiated tumors, blocking the pro-metastatic activity of the host and enhancing radiotherapy outcome.

## DISCUSSION

Radiotherapy is one of the conventional treatment modalities for cancer either as a monotherapy or accompanied by surgery and/or chemotherapy. It is an integral treatment protocol of locally advanced colorectal cancer, which was proven to significantly contribute to local control but marginally improves overall survival [30, 31]. However, in addition to the therapeutic benefits of radiotherapy, several clinical trials suggest that under certain circumstances radiotherapy promotes tumor growth and metastasis spread [32–34]. The exact mechanism for this is not fully understood, and is mostly attributed to activation of signaling pathways within the tumor that lead to accelerated proliferation [35]. Early radiobiology models of tumor repopulation in response to radiotherapy consider the tumor cells to be independent, but innovative models suggest that the microenvironment, namely, “tumor niches”, should be taken into account when estimating the repopulation phenomena [36]. Moreover, the signals released by the “dying” tumor cells induce different host responses, which are mostly described as inflammatory responses, and may also contribute to tumor cell regeneration [35]. In this study we sought to address how radiotherapy may contribute to metastasis, with a special emphasis on its effect on host cells that in turn generate pro-metastatic effects.

The contribution of anti-cancer therapy to tumor angiogenesis has been shown following both chemotherapy and radiotherapy [6, 37]. EPCs and other myeloid cells colonize treated tumors and support its re-growth following radiotherapy [6]. Radiation induces anti-angiogenic effects in tumors by directly promoting endothelial cell apoptosis [38], but at the same time, it indirectly promotes pro-angiogenic activity by the secretion of pro-angiogenic molecules such as TGF-β, bFGF, IL-10 and IL-5 in adjacent tissues enabling endothelial cell invasion, survival and angiogenesis [18, 39, 40]. In this study we focused solely on host pro-angiogenic effects following radiotherapy using non-tumor bearing mice. We found that HUVEC tube formation and microvessel sprouting from aortic rings did not significantly change in the presence of plasma from irradiated mice when compared to plasma from control mice. However, MDSCs which are known to support angiogenesis [41], were found in high numbers in Matrigel plugs containing plasma from irradiated mice as compared to that of control mice. These findings support studies showing that tumor re-growth following local radiation depends on vasculogenesis but not local angiogenesis [6, 15]. In contrast, other studies have indicated that direct radiation in doses lower than 2 Gy promotes endothelial cell migration, and induces tumor growth by enhancing angiogenesis in part due to the secretion of proangiogenic factors by the hypoxic irradiated tumors [21, 40].

A growing body of literature suggests that metastasis initiation in response to radiation is a consequence of a direct effect of radiotherapy on the tumor microenvironment affecting both tumor and stromal cells [16, 19]. In addition, it has been demonstrated that CYR61 and αVβ5 integrin are proteins that can promote invasion and metastasis of tumors grown in pre-irradiated sites [42]. This suggests that the reaction of stromal cells to irradiation can increase the pro-metastatic properties of tumor cells. In our study, we demonstrated that in addition to stromal cells at the irradiated site [42], plasma from irradiated mice also exhibits pro-metastatic properties as it induces invasion and migration of colon adenocarcinoma cells. Mice locally irradiated with a dose of 2 Gy prior to tumor inoculation succumb to metastasis before control untreated mice. These results suggest that pre-conditioning the mice with radiation can induce host effects which promote tumor cell aggressiveness. Consequently, an increase in metastases in liver and lungs was observed in orthotopic colon carcinoma tumor bearing mice that were exposed to a single dose of radiation. This study is in line with additional studies indicating that following irradiation the induction of BMDCs and angiogenesis can contribute to metastasis [19, 40].

Macrophages are known to be one of the major cell types that colonize primary tumors and contribute to metastasis [43, 44]. The pro-metastatic effect of macrophages is associated with an induction of tumor angiogenesis, an induction of local immunosuppression, or promotion of malignant cell invasion [45]. With respect to radiation, a previous study demonstrated that tumors with a high macrophage content are less responsive to radiotherapy than tumors with a low macrophage content [46]. Other studies have shown that radiation, on its own, can induce the accumulation of macrophages in the tumor bed, accompanied by migration and infiltration of neutrophils in the irradiated tissue [18]. Macrophages within the irradiated tumors have been shown to undergo activation leading to increased production of H_2_O_2_, lysosomal activity, and phagocytosis. The secretion of cytokines such as IL-1β and TNFα in the irradiated tumor microenvironment, can affect macrophage function and phenotype (for review see [47]). While in our study we show that a single dose of local radiation controlled primary tumor growth, it has been demonstrated that low-dose radiation promotes infiltration of activated T cells and pro-inflammatory macrophages to the treated tumor site, contributing to primary tumor growth retardation [48]. However, based on our study, the macrophages that home to irradiated tumors promote tumor cell aggressiveness and metastatic phenotype. It could be that the secretion of cathepsins and MMP9 from several types of BMDCs in response to chemotherapy [12, 49] may also hold in the case of radiation, and therefore can contribute to the dissemination of tumor cells from the primary tumor site, and the metastatic process. Indeed, when macrophages were depleted from mice that underwent radiotherapy, metastatic lesions were eliminated, suggesting a significant role for macrophage-induced metastasis at the irradiated tumor site.

DECA-14 is an analogue of dequalinium compounds that were widely used as antimicrobial agents and later studied for their anti-cancer effects. The compounds differ by the length of the hydrocarbon chain that links two aromatic moieties. DECA-14 contains a 14-carbon linker and when compared with analogues with shorter linkers such as DECA-10, was shown to inhibit PKC activity with maximum potency in mouse B16 melanoma cells [27]. Inhibition of PKC by DECA-14 in these cells consequently affected cellular pathways that control adhesion and migration [26, 27, 29]. In addition, DECA analogues such as DECA-10 can be selectively accumulated by a variety of cancer cells in their mitochondria [22, 23], and DECA-14 has been shown to specifically target tumor initiating cells [50]. In this study we showed that DECA-14 effectively abolishes radiation-induced metastasis. We demonstrated that DECA-14 may have anti-tumor activity not only by direct effects on tumor cells as previously shown [22, 28, 29, 50], but also by inhibiting motility, invasion and migration of host cells, in particular macrophages which are known to promote metastasis. The administration of DECA-14 with local radiation significantly inhibits the infiltration of pro-metastatic macrophages to the irradiated tumors, and therefore blunts the host pro-tumorigenic and pro-metastatic effects of radiation.

Overall, our results suggest that radiation, in addition to its therapeutic effects on tumor cells, may also have pro-angiogenic and pro-metastatic effects which can explain enhanced tumor regrowth and metastasis following therapy. In this regard, it is important to note that in our experiments the mice were exposed to one dose of 2 Gy radiation. In the clinic, patients are exposed to multiple doses of radiation in a fractionation therapy schedule reaching a total dose of approximately 60–80 Gy, when 2 Gy dose is usually the maximum dose per day. Therefore, enhanced metastasis due to host effects in response to radiation should be further tested in the clinical setting. However, our results provide some additional insights into the pro-tumorigenic effects of radiation. Blocking the pro-tumorigenic effects generated by macrophages, for example, may result in enhanced therapeutic outcome of radiotherapy.

## MATERIALS AND METHODS

### Cell culture

HUVECs, purchased from Lonza (USA), were cultured in M199 medium supplemented with 20% fetal calf serum (FCS) and 50 mg/ml BT-203, an endothelial mitogen factor. Human colon cancer cells (HCT116, HT29 and SW480) and murine macrophages (J774), purchased from the American Type Culture Collection (ATCC), were cultured in Dulbecco's modified Eagle's medium (DMEM) supplemented with 10% FCS. CT26 murine colon cancer cells (ATCC) were cultured in RPMI medium supplemented with 10% FCS. All cells were cultured in the presence of 1% penicillin-streptomycin, 1% sodium pyruvate, and 1% L-glutamine at 37°C in a 5% CO_2_ environment for no more than 6 months after being thawed from original stocks.

### Animal models, treatments, and live imaging

The use of animals and experimental protocols were approved by the Animal Care and Use Committee of the Technion. An orthotopic colon carcinoma model was generated as previously described [51]. Briefly, HT29, SW480, HCT116, and CT26 murine or human cells were subcutaneously implanted in 8–10 week old CB.17 female SCID mice (for HT29, SW480, and HCT116) or female BALB/c mice (for CT26). When tumors reached a size of 500–750 mm^3^ they were resected and kept sterile. Tumors were chopped into small fragments (1–3 mm^3^), and were subsequently sutured to the cecum of naïve mice. At end point (usually within 2–6 weeks), mice were sacrificed and lungs, liver, colon, and lymph nodes were examined for the presence of macro-metastases. In some experiments tumor size was monitored regularly by IVIS™ imaging system when using luciferase-tagged tumor cells. Four weeks after tumor implantation, mice were exposed to a single dose of 2 Gy local radiation in the tumor area in the abdominal cavity. Four weeks later mice were sacrificed. Livers and lungs were removed and processed histologically in order to detect metastases. For depletion of macrophages, mice were injected intraperitoneally with 200 μl of liposomal chlodronate or control liposomes (Encapsula NanoSciences, Brentwood, TN) 24 hours before exposure to 2 Gy radiation, as per the manufacturer's instructions. For the experimental pulmonary metastasis model, CT26 cells (5 × 10^4^) were injected intravenously to the tail vein of 8–10-week-old female BALB/c mice and survival was monitored. In some experiments, as indicated in the text, mice were intraperitoneally injected with 2.5 mg/kg dequalinium-14 (DECA-14) synthesized as described in [27].

Bioluminescent imaging of luciferase-expressing tumors was performed with a highly sensitive, cooled charge coupled device (CCD) camera mounted in a light-tight specimen box (IVIS; Xenogen Corp., Waltham, MA). Briefly, mice were injected intraperitoneally with substrate D-luciferin at 150 mg/kg, and after a 10-minute interval, they were anesthetized using isoflurane. Mice were then placed onto the warmed stage inside the light-tight camera box, with continuous exposure to isoflurane (EZAnesthesia, Palmer, PA) for maintenance of anesthesia. The mice were imaged for 1 minute. Light emitted from the bioluminescent cells was detected by the IVIS camera system with images quantified for tumor burden using a log-scale color range set at 1 × 10^6^ to 1 × 10^8^ and measurement of average radiance per second (photons per second) using Living Image software (Xenogen).

### Irradiation

Mice were locally irradiated to the abdominal cavity or tumor area with a linear accelerator 6 MeV electron beam using Elekta Precise (ElektaOncology Systems) at a dose rate of 40 cGy per minute, for a total dose of 2 Gy, 6 Gy or 10 Gy at room temperature (Department of Radiation Therapy, Rambam Medical Center, Haifa, Israel), as indicated in the text.

### Condition medium from macrophages and bone marrow derived cells

BMDCs were flushed from the femurs of 8–10 week old BALB/c mice 24 hours after they were either exposed to 2 Gy local radiation, or left alone (control group). BMDCs were cultured in serum-free DMEM for 48 hours in a concentration of 1 × 10^6^ cells/ml. Conditioned medium was then collected and stored in −80°C. To obtain macrophages, 8–10 week old BALB/c mice were injected intraperitoneally with 3 mL 4% thioglycollate solution (BD Biosciences, USA). Two days later, mice were exposed to 2 Gy local radiation or left untreated. After an additional 24 hours, mice were euthanized and peritoneal lavage was performed. Peritoneal macrophages were then cultured with serum-free DMEM for 48 hours in a concentration of 1 × 10^6^ cells/ml, in order to obtain conditioned medium. The conditioned medium was stored at −80°C until further used.

### Modified Boyden chamber assay

The invasion and migration properties of HUVECs, macrophages, and colorectal tumor cells were evaluated in either Matrigel- or fibronectin-coated Boyden chambers, using a previously described protocol [12, 52]. Briefly, serum-starved cells (2.5 × 10^5^ cells in 0.2 ml medium) were added to the filter that was coated with either Matrigel (BD Biosciences, USA) for invasion or fibronectin (10 μg/ml) for migration. The lower compartment was filled with serum-free DMEM supplemented with 10% plasma (and 5% plasma in the case of HUVECs) obtained from mice exposed to 2 Gy radiation or untreated mice. In the case of macrophages, serum-starved cells were placed in the upper compartment of the Boyden chamber in medium supplemented with 100 nM of dequalinium-14 (DECA-14), as previously shown [50]. The lower compartment was filled with serum-rich DMEM. After 24 hours, the cells that migrated to the bottom filter, were stained with Crystal violet and counted under an inverted microscope (Lieca DMIL LED) per x100 objective-field. All experiments were performed in triplicate.

### MMP2/9 detection by gelatin zymography

Samples of plasma and conditioned medium of BMDCs and abdominal macrophages obtained from locally irradiated mice or respective controls were evaluated for MMP2/9 activity as previously described [12]. The samples were resolved on 10% acrylamide Ready Gel Zymogram Gels (Bio-rad, Israel) under non-reducing conditions. The gels were incubated in 2.5% Triton X-100 for 1 hour and transferred to a solution containing 50 mM Tris, 0.2 M NaCl, 5 mM CaCl_2_ at pH 7.6 for 16 hours at 37°C. Subsequently, gels were stained with 0.5% Coomassie Blue for one hour and then destained in methanol/acetic acid/H_2_O (10:10:80). Intensities of the bands were quantified by densitometry, using TotalLab Quant software (TotalLab, UK). Experiments were performed in triplicate, and the average of densitometry analyses was included.

### Matrigel plug assay

Matrigel (0.5 mL) containing 10% plasma from control or locally-irradiated BALB/c mice was injected subcutaneously into each flank of BALB/c mice (*n* = 5 mice/group). The plugs were removed 10 days later and processed for immunohistochemical evaluation or flow cytometry analysis as detailed below.

### Evaluation of BMDCs by flow cytometry

Whole blood was obtained from anaesthetized mice by retro-orbital sinus bleed. Matrigel plugs or tumors were prepared as single cell suspensions. Cell suspensions were immunostained with antibody mixtures to detect cell types defined by their surface markers as follows: EPCs: CD31^+^/VEGFR2^+^/CD117^+^/CD45^−^; hemangiocytes: CXCR4^+^/VEGFR1^+^/CD45^+^; MDSCs: Gr-1^+^/CD11b^+^/CD45^+^; mast cells: FCεR1^+^/CD34^+^/CD117^+^/Gr-1^−^/CD45^+^ and macrophages: F4/80^+^, CD11b^+^, Gr-1^−^, CD115^+^. All monoclonal antibodies were purchased from Biolegend, BD Biosciences or R&D systems and used according to the manufacturers' instructions. The monoclonal antibodies were purchased conjugated with the following fluorochromes: Gr-1-Fluorescein isothiocyanate (FITC), VEGFR2-Phycoerythrin (PE), CXCR4-PE, CD11b-Peridinin-chlorophyll proteins (PerCP), CD31-FITC, CD117-Allophycocyanin (APC), VEGFR1-APC, and CD45-APC-Cy7, FCεR1-PE, F4/80-PE, CD115-APC, CD34-eFluor450. When necessary, after red blood cell lysis, cell suspensions were analyzed by CyAn ADP Flow cytometer and Summit v4.3 software (Beckman Coulter). An acquisition of at least 100,000 cells per sample was performed. Analyses were considered informative when an adequate number of events (typically 50–150) were collected in the EPC, hemangiocyte, MDSC, mast cell, or macrophage enumeration gates in untreated control animals. Percentages of stained cells were determined and compared with appropriate negative controls. Positive staining was defined as being greater than non-specific background staining, and 7-aminoactinomycin D (7AAD) was used to distinguish apoptotic and dead cells from viable cells.

### Aortic ring sprouting assay

Aortic ring assay was performed as previously described [53]. Briefly, 1 mm long aortic rings from non-tumor bearing BALB/c mice were embedded in Matrigel (BD Bioscience), and then cultured in serum-free DMEM supplemented with 10% plasma from control or locally irradiated mice for 10 days. Endothelial cell growth supplement (ECGS) mitogen (BT-203, 20 μg/ml) (Biomedical Technologies Inc, Stoughton, MA) was used as a positive control. The sprouting microvessels observed in the Matrigel-embedded aortic rings were counted under light-microscopy per x10 objective field (magnification X100). All experiments were performed in triplicate.

### Tube forming assay

Tube formation on Matrigel was carried out as previously described [54]. Briefly, HUVECs were seeded in Matrigel-coated 48-well tissue culture plates (2 × 10^4^ cells/well) and incubated in 5% FCS M-199 medium supplemented with 10% plasma from mice, 24 hours after they were exposed to 2 Gy radiation in the abdominal cavity. Phase-contrast images of microvessel tubes were captured after 4 hours at 100x magnification using the Leica CTR 6000 (Leica Microsystems). The images were analyzed using ImageJ software.

### Scratch wound assay

The scratch wound assay was performed as previously described [55]. Briefly, J774 murine macrophages were cultured in culture-inserts (Ibidi, Germany) in DMEM supplemented with 10% FCS. The cells were starved in serum-free DMEM for 16 hours before they were assayed. Inserts were removed and the cells were incubated in serum-free DMEM supplemented with 10% plasma from untreated or DECA-14-treated (2.5 mg/kg) mice. Time-Lapse images of cell migration were captured using Zeiss Axio Observer inverted fluorescent microscope (Zeiss, Germany), per x5 objective-field and analyzed with ImagePro Premier software (Media Cybernetics, Rockville, MD).

### Analysis of epithelial-to-mesenchymal transition (EMT)

Immunohistochemistry and Western Blot analyses of EMT were performed as previously described, using SW480 cells [25]. Briefly, SW480 cells were cultured on glass coverslips (for immunohistochemistry) or in 60 mm plates (for Western Blot). The cells underwent serum-starvation for 24 hours, after which they were cultured in the presence of conditioned medium of abdominal macrophages obtained from control and irradiated mice. For immunohistochemistry, after 24 hours, cells were fixed with 4% paraformaldehyde and immunostained for epithelial (E-cadherin) and mesenchymal (vimentin) specific markers using monoclonal anti-E-cadherin and polyclonal anti-vimentin primary antibodies (1:100, Santa-Cruz Biotechnology) and Cy3-conjugated goat anti-mouse and DyLight-488-conjugated goat anti-rabbit secondary antibodies (1:200, Jackson ImmunoResearch, PA, USA). Nuclei were counterstained with DAPI. Images were captured using the Leica CTR 6000 microscope system (Leica Microsystems). For Western Blot, after 24 hours, cell lysates were separated by 10%SDS-PAGE and electrotransferred to nitrocellulose membranes. Immunostaining was performed using the antibodies indicated above. GAPDH served as a loading control. Membranes were incubated with HRP-conjugated goat anti-mouse and goat-anti-rabbit secondary antibodies. Intensities of the bands were quantified by densitometry, using TotalLab Quant software (TotalLab, UK). Experiments were performed in triplicate, and the average of densitometry analyses was included.

### Histology processing and immunostaining

Tumors, lungs and livers collected from irradiated or control mice were embedded in OCT (Tissue-Tek, Sakura Finetek USA Inc., USA) and stored at −80°C. Matrigel plugs were fixed in 4% paraformaldehyde at room temperature for 24 hours, and subsequently embedded in OCT at 4°C for 48 hours before they were frozen at −80°C. Matrigel plugs and organs were cryosectioned and stained with hematoxylin and eosin (H&E) to assess colonization of host cells and identify metastatic lesions, respectively. Tumors and organs were immunostained with rat anti-mouse F4/80 antibody already conjugated to Phycoerythrin (1:200, BD Biosciences) to detect macrophages, rat anti-human HLA (1:200, BD Biosciences) and a secondary Cy3 anti-rat (1:200, Jackson ImmunoResearch, PA, USA) to detect human cells, or rat anti-mouse CD31 antibody (1:200, BD Biosciences), and a secondary Cy3 anti-rat (1:200, Jackson ImmunoResearch) to detect endothelial cells. Nuclei were stained with DAPI. Stained sections were visualized using a Leica CTR 6000 microscope system (Leica Microsystems). The microvessel density was calculated as the number of vessel structures per field (*n* > 10 fields/group).

### Protein Kinase C (PKC) activity assay

Eight-to-ten week old BALB/c mice were injected with 3 ml of 4% thioglycolate solution to the abdominal cavity. After 72 hours, abdominal macrophages were collected and washed with PBS. PKC activity in macrophages was evaluated using MESACAP protein kinase assay kit (MBL, Japan). All experiments were performed in triplicate.

### Statistical analysis

Data are expressed as means ± SD, and the statistical significance of differences was assessed by one-way ANOVA, followed by Newman-Keuls ad hoc statistical test or student *t*-test using GraphPad Prism 5 software (La Jolla, CA). The Fisher Exact Test was used for calculating the probability of metastasis in the *in vivo* experiments when comparing control and irradiated mice. For the calculation of mouse survival, a Kaplan-Meier Survival Curve statistical analysis was performed in which the uncertainty of the fractional survival of 95% confidence intervals was calculated. Differences between all groups were compared with each other or were compared to control (in the case of *t*-test), and were considered significant at values of *P < .05, **P < .01, and ***P < .001.

## SUPPLEMENTARY FIGURES AND TABLES


