# Supplementary material for: Dequalinium blocks macrophage-induced metastasis following local radiation
Source: Oncotarget. 2015 Aug 6;6(29):27537–54. doi: 10.18632/oncotarget.4826 (PMC4695007; doi:10.18632/oncotarget.4826)
Supplement: Supplementary file 1 [file oncotarget-06-27537-s001.pdf]

## SUPPLEMENTARY FIGURES AND TABLES

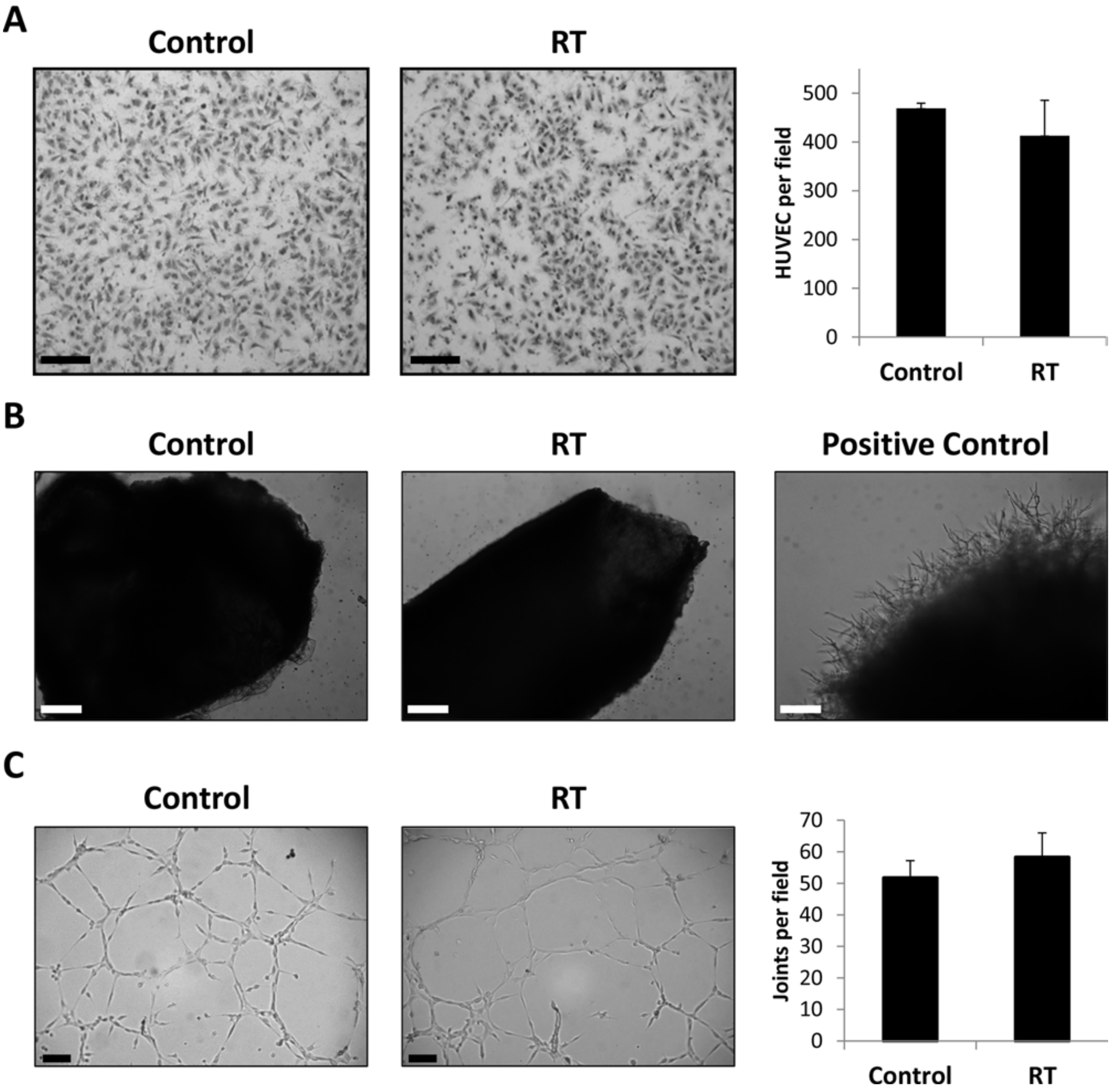


**Supplementary Figure S1: Endothelial cell activity is not altered in the presence of plasma from locally irradiated mice.** Medium containing 10% plasma from control mice or from mice 24 hours after they were exposed to 2 Gy RT (RT) was applied to HUVEC modified Boyden chambers to assess migration **A;** microvessel sprouting from aortic rings (Scale bars = 200 µm) **B;** or HUVEC tube formation (Scale bars = 200 µm) **C;** as described in Materials and Methods (Scale bars = 100 µm). ECGS (BT-203) (20 µg/ml) was used as a positive control in the aorta ring sprouting assay.

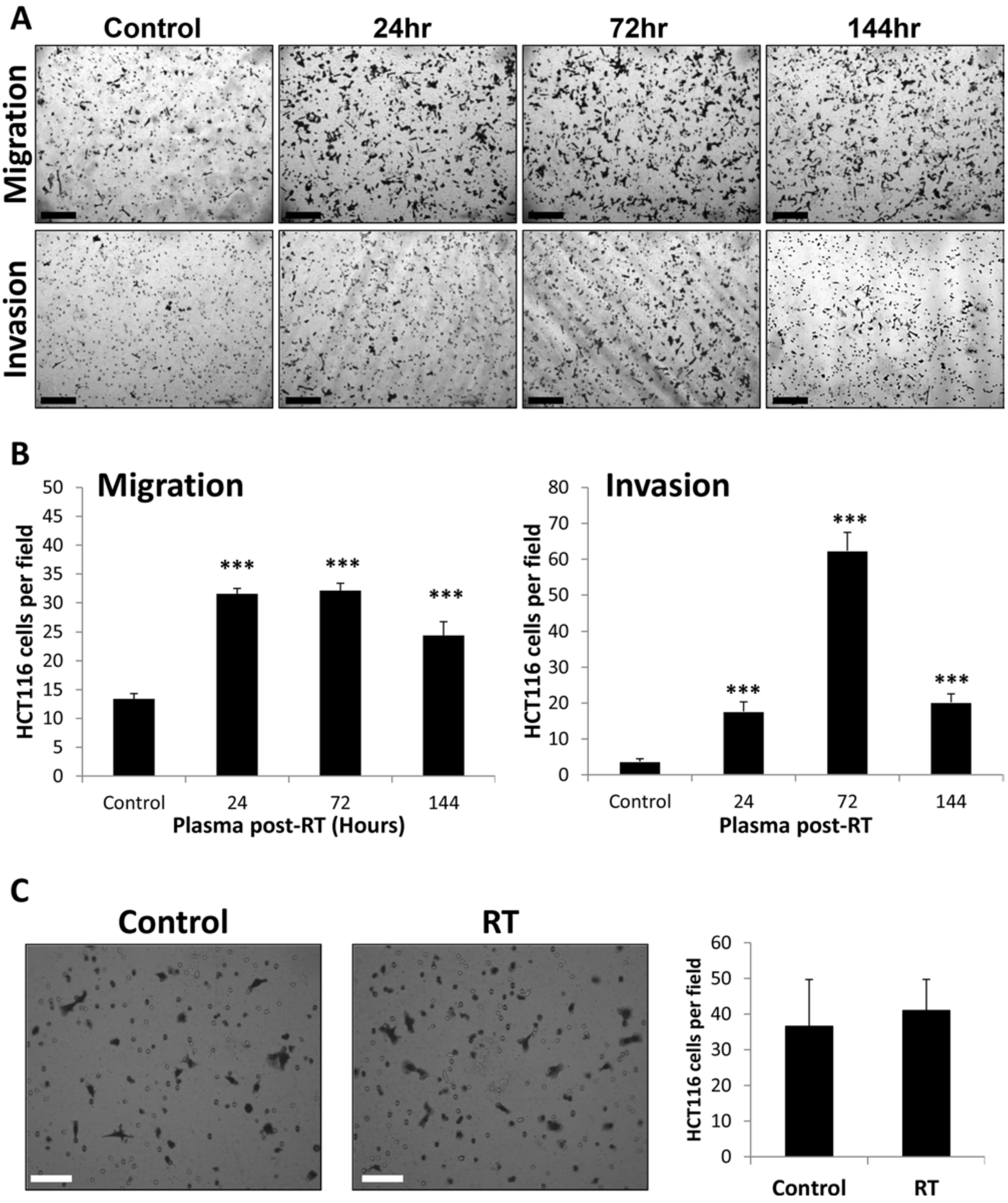


**Supplementary Figure S2: Plasma from locally irradiated mice promotes migration and invasion of murine colon cancer cells. A-B.** Eight-to-ten week old BALB/c mice were exposed to single dose 2 Gy RT in the abdominal cavity. Plasma was collected at baseline (Control), 24, 72 and 144 hours after radiation. (A) The invasion and migration properties of HCT116 human colon carcinoma cells were assessed using the Boyden chamber assay, and the percentage of cell coverage on the membrane was quantified (B). Scale bars = 200 μm. In a parallel experiment the plasma was applied to the upper compartment of the modified Boyden chamber and tumor cell motility was evaluated **C.** Scale bars = 100 μm. ***$p < 0.001$.

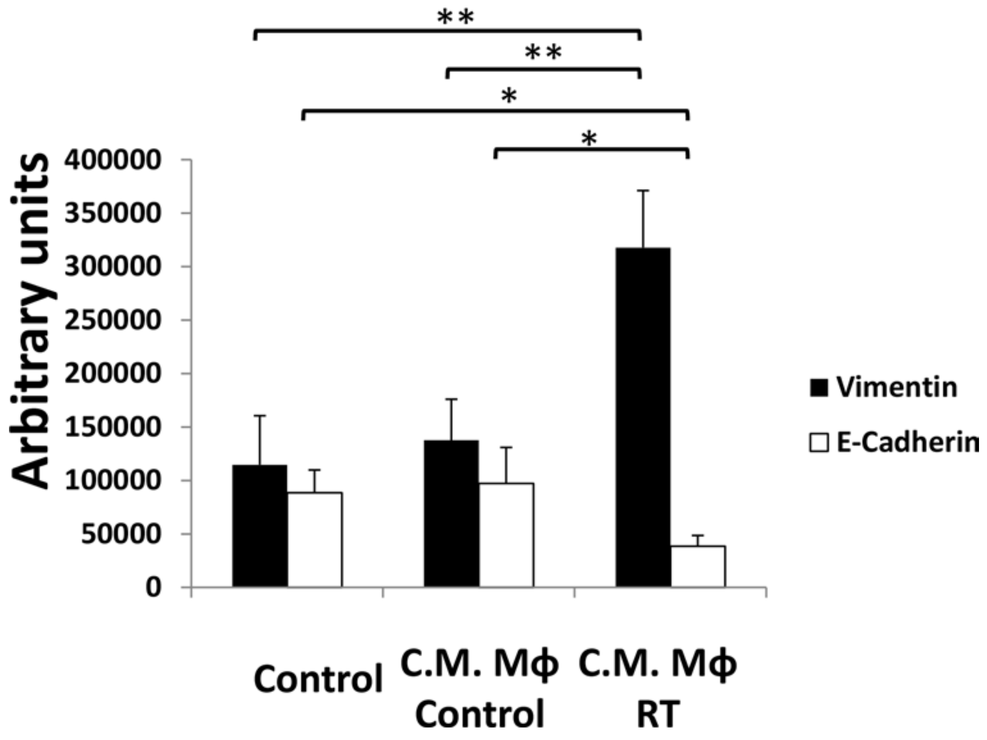


**Supplementary Figure S3: Macrophages from irradiated mice promote epithelial-to-mesenchymal transition.** To determine the extent of epithelial-to-mesenchymal transition (EMT), SW480 cells were incubated with conditioned medium of macrophages from control mice (CM Mφ Control) or from irradiated mice (CM Mφ RT). As a control, cells were cultured in DMEM+10% FCS. Cell lysates were analyzed by Western Blot. The expression levels of E-cadherin and vimentin were determined by densitometry analysis of the Wester Blots. Three independent experiments were performed.

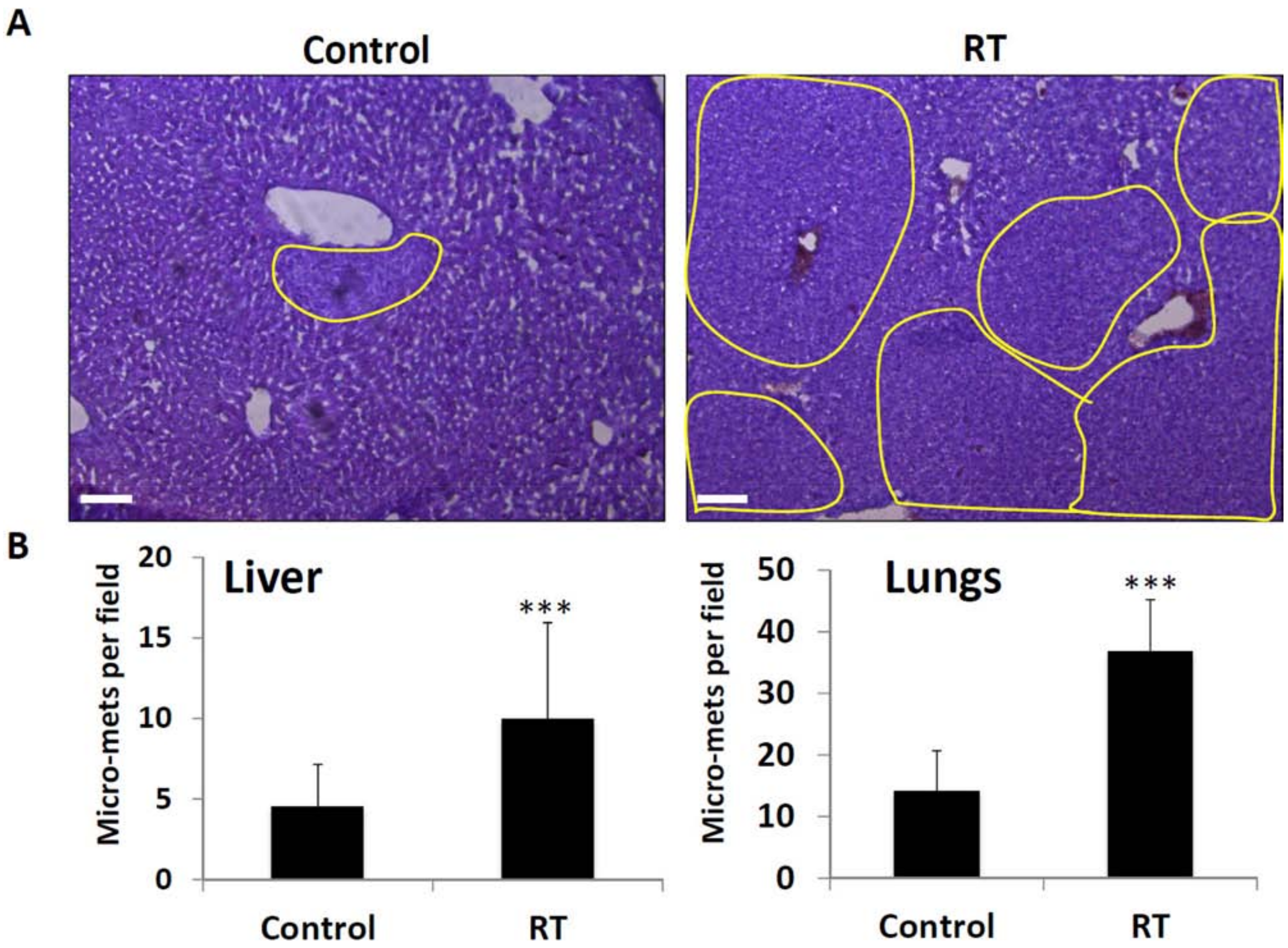


**Supplementary Figure S4: Radiotherpy-induced metastasis in mice bearing orthotopic CT26 colon cancer.** Eight-to-ten week old BALB/c mice were orthotopically implanted with CT26 tumors, similar to the SW480 tumor model explained in Figure 4 ($n = 5$ mice/group). After 2 weeks, the mice were either irradiated in the abdominal cavity (RT) at a dose of 2 Gy or left untreated (Control). At end point (one week after therapy), liver and lungs were removed and examined for metastases. **A.** Liver sections were stained with H&E. Yellow circles represent metastatic lesions. Scale bars = 100 μm. **B.** The quantification of lung and liver metastases is presented. ***$p < 0.001$.

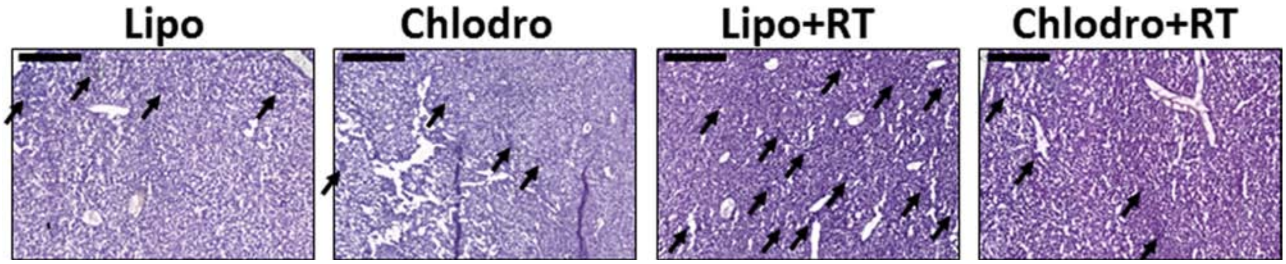


**Supplementary Figure S5: Macrophage-depleted irradiated mice exhibit reduced liver micro-metastasis.** Eight-to-ten week old SCID mice were orthotopically implanted with SW480 tumors. After 4 weeks, the mice were either injected with empty liposomes (Lipo) or liposomes containing Clodronate to deplete macrophages (Clodro). After 24 hours, mice were either exposed to 2 Gy radiation (RT) in the tumor area or left untreated (Control) (*n* = 5 mice/group). After an additional 4 weeks the mice were sacrificed and the liver was processed for histopathology analysis. Liver sections were stained with H&E. Black arrows represent micrometastatic lesions. Scale bars = 500 μm.

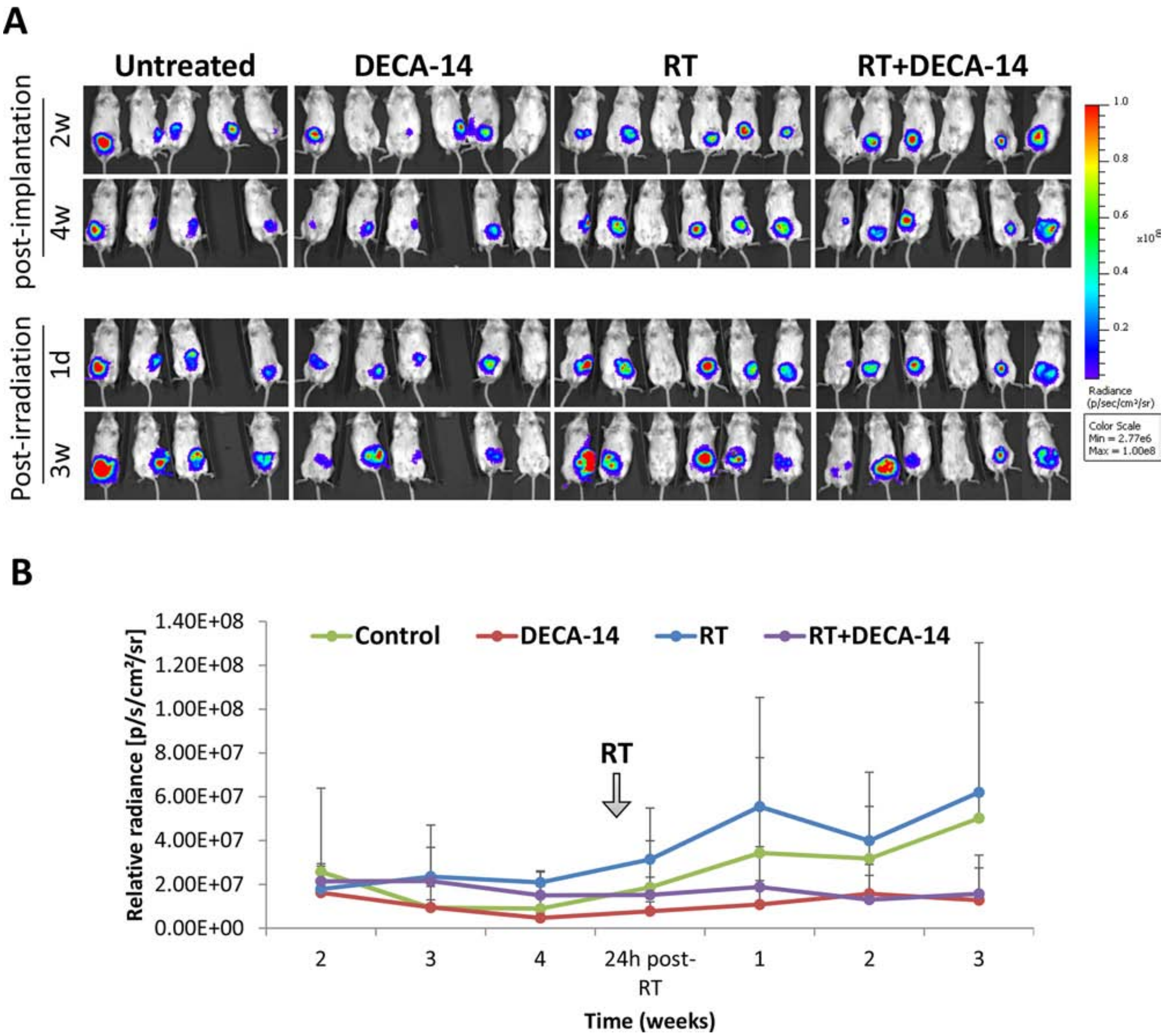


**Supplementary Figure S6: DECA-14 inhibits SW480 orthotopic colon carcinoma tumor growth.** Eight-to-ten week old SCID mice were orthotopically implanted with SW480 tumors. After 4 weeks, mice were either left untreated or injected with 2.5 mg/kg DECA-14, and 24 hours later, mice were either irradiated in the tumor area (RT) or left untreated (Control). IVIS images were captured during the experiment as indicated in the Figure **A,** and a graph summarizing the quantification of tumor illumination is presented **B.**

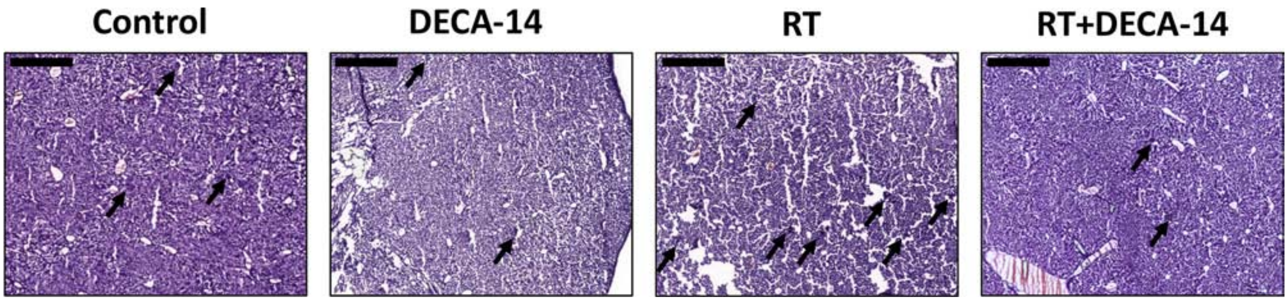


**Supplementary Figure S7: Dequalinium-14 (DECA-14) inhibits radiation-induced liver micro-metastasis.** Eight-to-ten week old SCID mice were orthotopically implanted with SW480 tumors. After 4 weeks, the mice were peritoneally injected with 2.5 mg/kg DECA-14, and after 24 hours, mice were either irradiated in the tumor area (RT) or left untreated (Control) ($n$ = 5–6 mice/group). Four weeks later, the mice were sacrificed, and lungs and liver were removed and processed for histopathology analysis. Liver sections were stained with H&E. Black arrows indicate micrometastatic lesions. Scale bar = 500 µm.

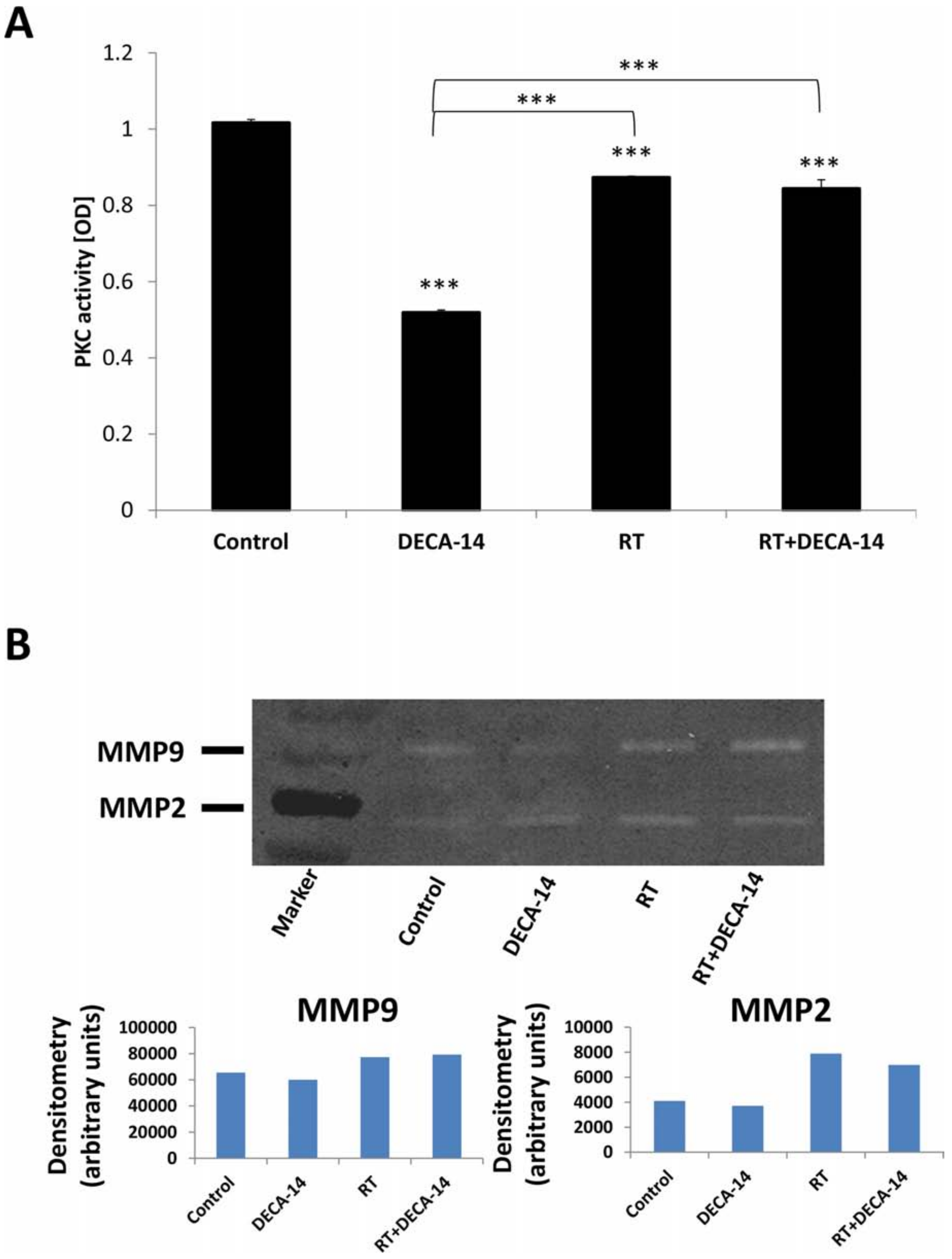


**Supplementary Figure S8: PKC and MMP9/2 activities in macrophages obtained from irradiated and/or DECA-14-treated mice.** Abdominal macrophages obtained by thioglycolate induction were collected from 8–10 week old BALB/c mice, 24 hours after they were exposed to RT with or without DECA-14 administration. **A.** PKC activity in macrophages was evaluated as described in Materials and Methods. **B.** The macrophages were cultured in serum-free DMEM for 48 hours in a concentration of $1 \times 10^6$ cell/ml, and levels of MMP9 and MMP2 activity was evaluated by zymography followed by densitometry analyses. ***$p < 0.001$.

**Supplementary Table S1: The metastatic ability of human and murine colon cancer cell lines orthotopically implanted in mice**

| Cell line | Source | Orthotopic colon model | Invasion |
|---|---|---|---|
| HT29 | Human | + | Metastatic (colon) |
| SW480 | Human | + | Rarely metastasize |
| HCT116 | Human | + | Metastatic (colon/liver) |
| CT26 | Murine | + | Metastatic (colon/liver/lungs/lymph nodes) |

HT29, SW480, HCT116, and CT26 murine or human cells (as indicated in the table) were subcutaneously implanted into the flanks of SCID (for HT29, SW480, and HCT116) or BALB/c (for CT26) mice. When tumors reached a size of 500–750 $mm^3$ they were resected and kept sterile. Tumors were chopped into small fragments (1–3 $mm^3$), and were subsequently sutured to the cecum of naïve mice, and tumor growth was assessed. At end point (usually within 6 weeks), mice were sacrificed and lungs, liver, colon, and lymph nodes were examined for the presence of macro-metastases.

**Supplementary Table S2: The incidence of liver and lung macro-metastases in SW480 bearing mice exposed to local radiation**

| Experiment | Control (macrometastasis) | | RT (2 Gy) (macrometastasis) | |
|---|---|---|---|---|
| | Liver | Lungs | Liver | Lungs |
| 1 | 1 | 0 | 1 | 2 |
| | (1/6) | | (3/6) | |
| 2 | 1 | 1 | 2 | 4 |
| | (2/6) | | (6/6) | |
| 3 | 0 | 0 | 0 | 1 |
| | (0/3) | | (1/3) | |
| 4 | 0 | 0 | 0 | 2 |
| | (0/6) | | (2/6) | |
| 5 | 0 | 0 | 1 | 3 |
| | (0/4) | | (4/4) | |
| **Total** | **3 of 25 (12%)** | | **16 of 25 (64%)** | |

SW480 tumors were orthotopically implanted in the cecum of 8–10 week old CB.17 SCID mice. After 4 weeks, the mice were either irradiated in the abdominal cavity (RT) or left untreated (Control). At end point (additional 4 weeks), liver and lungs were removed and examined for macro-metastases. The number of mice bearing macro-metastasis in either liver or lungs is shown for each experiment. The incidence of metastasis (in both liver and lungs) per group of mice is indicated in brackets.
